# Ex Vivo and In Vitro Proteomic Approach to Elucidate the Relevance of IL‐4 and IL‐10 in Intervertebral Disc Pathophysiology

**DOI:** 10.1002/jsp2.70048

**Published:** 2025-02-10

**Authors:** Paola Bermudez‐Lekerika, Sofia Tseranidou, Exarchos Kanelis, Andrea Nüesch, Katherine B. Crump, Leonidas G. Alexopoulos, Karin Wuertz‐Kozak, Jérôme Noailly, Christine L. Le Maitre, Benjamin Gantenbein

**Affiliations:** ^1^ Tissue Engineering for Orthopaedics and Mechanobiology, Bone and Joint Program, Department for BioMedical Research (DBMR), Faculty of Medicine University of Bern Bern Switzerland; ^2^ Graduate School for Cellular and Biomedical Sciences (GCB) University of Bern Bern Switzerland; ^3^ Department of Engineering Universitat Pompeu Fabra Barcelona Spain; ^4^ Testing Services Protavio Ltd, Demokritos Science Park Athens Greece; ^5^ School of Mechanical Engineering National Technical University of Athens Zografou Greece; ^6^ Division of Clinical Sciences, School of Medicine and Population Health University of Sheffield Sheffield England; ^7^ Department of Biomedical Engineering Rochester Institute of Technology Rochester New York USA; ^8^ Spine Center, Schön Klinik München Harlaching Academic Teaching Hospital and Spine Research Institute of the Paracelsus Private Medical University Salzburg (Austria) Munich Germany; ^9^ Department of Orthopaedic Surgery and Traumatology, Inselspital, Bern University Hospital, Faculty of Medicine University of Bern Bern Switzerland

**Keywords:** immunohistochemistry, interleukin‐10, interleukin‐4, intervertebral disc, low back pain, secretome proteomics

## Abstract

**Background:**

This study investigates the native presence and potential anabolic effects of interleukin (IL)‐4 and IL‐10 in the human intervertebral disc (IVD).

**Methods:**

Human nucleus pulposus (NP) cells cultured in 3D from trauma and degenerate IVDs and NP explants were stimulated with 10 ng/mL IL‐4, IL‐10, or each in combination with 1 ng/mL IL‐1β stimulation. The role of IL‐4 and IL‐10 in the IVD was evaluated using immunohistochemistry, gene expression, and Luminex multiplex immunoassay proteomics (73 secreted) and phosphoproteomics (21 phosphorylated proteins).

**Results:**

IL‐4, IL‐4R, and IL‐10R expression and localization in human cartilage endplate tissue were demonstrated for the first time. No significant gene expression changes were noted under IL‐4 or IL‐10 stimulation. However, IL‐1β stimulation significantly increased *MMP3*, *COX2*, *TIMP1*, and TRPV4 expression in NP cells from trauma IVDs. Combined IL‐4 and IL‐1β treatment induced a significant increase in protein secretion of IL‐1α, IL‐7, IL‐16, IL‐17F, IL‐18, IFNγ, TNF, ST2, PROK1, bFGF2, and stem cell factor exclusively in NP cells from degenerated IVDs. Conversely, the secretome profile of explants revealed an IL‐4–mediated decrease in CXCL13 following treatment with IL‐1β. Combined IL‐10 and IL‐1β treatment increased neurotrophic growth factor secretion compared with IL‐10 baseline.

**Conclusions:**

The NP cell phenotype affects the pleiotropic role of IL‐4, which can induce a pro‐inflammatory response in the presence of catabolic stimuli and enhance the effects of IL‐1β in degenerated IVDs. Environmental factors, including 3D culture and hypoxia, may alter IL‐4's role. Finally, IL‐10's potential neurotrophic effects under catabolic stimuli warrant further investigation to clarify its role in IVD degeneration.

## Graphical abstract

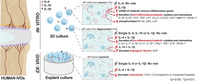

## 1 Introduction

Low back pain (LBP) is the leading cause of years lived with disability globally, with around 619 million cases reported in 2020 and females disproportionately affected [1]. The etiology of LBP is multifactorial, with intervertebral disc (IVD) degeneration accounting for ~40% of chronic LBP cases [2]. The IVD comprises three structures: The nucleus pulposus (NP), the surrounding annulus fibrosus (AF), and the cartilaginous endplates (CEP) covering the vertebral bodies. High proteoglycan concentration and physical pressure in the NP inhibit blood vessel ingrowth and isolate the NP from the host immune system [3]. Thus, IVD is immune‐privileged, but an immune response can be provoked if exposure occurs from degeneration or herniation [4, 5].

Interleukin (IL)‐4 and IL‐10 are pleiotropic anti‐inflammatory cytokines that primarily suppress the pro‐inflammatory milieu. Both their receptors (IL‐4R and IL‐10R) are expressed on immune and non‐immune cells [6, 7]. IL‐4 has been shown to have a protective role in chondrocytes by preventing proteoglycan loss following IL‐1β and tumor necrosis factor (TNF) stimulation [8] and inhibiting matrix metalloproteinase (MMP)‐13 induction after mechanical loading [9]. In fibroblasts, IL‐4 inhibits IL‐1–induced MMP‐3 expression through negative crosstalk at the c‐Jun N‐terminal kinase activation level, thereby maintaining a balance between pro‐inflammatory and anti‐inflammatory processes [10]. It also reduces neutrophil migration and promotes immune cell signaling through STAT6 [11]. Overexpression of IL‐10 in articular chondrocytes upregulates *COL2A*, downregulates *MMP‐13* expression, and prevents *ACAN* downregulation after TNF stimulation [12]. In addition, IL‐10 suppresses chemokines such as C‐C motif chemokine ligand (CCL) 2, CCL3, and C‐X‐C motif ligand (CXCL) 8, limiting leukocyte infiltration [13, 14], and reduces the release of pro‐inflammatory cytokines: TNF, IL‐1β, IL‐6, and interferon (IFN)‐γ in macrophages and dendritic cells [13, 14]. IL‐10 also plays a role in muscle repair by modulating the immune response, reducing inflammation, and promoting regeneration of muscle fibers after injury [15]. Together, IL‐4 and IL‐10 limit inflammation and promote tissue repair.

In IVD tissue, IL‐4 decreased the induction of IL‐6 and IL‐8 by lipopolysaccharide [16]. Similarly, IL‐10 decreased the inhibitory effects of IL‐1β on *COL2A1* and *ACAN* gene and protein expression in a rat in vivo model [17]. IL‐10 also inhibited p38 mitogen‐activated protein kinase (MAPK) signaling pathway in human NP cells [17]. Furthermore, a recent novel in silico regulatory network model of NP cells highlighted the potential importance of these two anti‐inflammatory cytokines [18]. However, the precise roles of IL‐4 and IL‐10 in the IVD remain unclear.

This study aimed to test the hypothesis that IL‐4 and IL‐10 are key anabolic factors in the IVD and can rescue NP cells from a catabolic environment induced by IL‐1β. Moreover, different NP cell phenotypes (from trauma and degenerated IVDs), culture systems (in vitro alginate or ex vivo explant), and osmolarity environments were investigated to determine the role of IL‐4 and IL‐10 in the NP. Specifically, the influence on gene expression profiling, immunohistochemistry (IHC), and secretomic and phosphoproteomic analysis was investigated.

## 2 Materials and Methods

### 2.1 Human NP Cell Isolation

Human IVD tissues were collected from female and male patients undergoing surgery with informed consent (Sheffield Research Ethics Committee [IRAS‐10266] and general ethical consent of the Insel University Hospital of Bern, Switzerland) (Table 1). Human traumatic IVD tissues from patients aged between 31 and 60 years old (44.42 ± 10.41[mean ± SD]) and degenerated IVD tissues from patients aged between 44 and 78 years old (55.6 ± 15.79 [mean ± SD]) were collected. Human IVD tissue fragments were washed twice with phosphate buffered saline (PBS) and morphologically separated into NP and AF tissues. Human NP cells from trauma IVDs were isolated by 1 h 1.9 mg/mL pronase ([7 U/mg], Roche Diagnostics, Basel, Switzerland) digestion, followed by overnight digestion on a 600 rpm orbital shaker at 37°C with 0.23 mg/mL collagenase II ([285 U/mg] Worthington, London, UK) in serum‐free high‐glucose (4.5 g/L) Dulbecco's Modified Eagle Medium (HG‐DMEM, Invitrogen, Paisley, UK) containing 1% v/v penicillin/streptomycin (P/S) at 20 mL/g. NP cells from degenerated IVDs were isolated by four‐hour digestion of human NP tissue fragments with 0.5 mg/mL collagenase type II ([64 U], #17101–015, Sigma‐Aldrich, Gillingham, UK) in serum‐free HG‐DMEM containing 1% v/v P/S at 20 mL/g on an orbital shaker at 37°C. The remaining tissue was removed by 70 or 100 μm cell strainer filtration (#352360, FalconTM, Thermo‐Fisher Scientific, Basel, Switzerland), and cell number and viability were determined by trypan blue. Following isolation, human NP cells were expanded until passage 3 in monolayer in HG‐DMEM supplemented with 10% v/v heat‐inactivated FCS (Invitrogen), 1% v/v P/S, 2,5 μg/mL Amphotericin B, 25 μg/mL L‐Ascorbic acid 2‐phosphate sesquimagnesium salt hydrate (A8960, Sigma‐Aldrich) (NP expansion media) [19] and maintained at 37°C in a humidified atmosphere containing 5% O_2_, 5% CO_2_. All T‐flasks (including NP cells from trauma or degenerated IVDs) were trypsinized when cell confluency was nearly reached (80%–90%).

**TABLE 1:** Patient details of human IVD samples used in this study.

Ref	Diagnosis	Gender	Age	IVD level	Intact IVD?	Culture system/processing	Cytokine treatment	IHC	Proteomic analysis
1	Radicular pain	F	32	L4/5	Y	Explant	Y	N	Y
2	Radicular pain	F	56	C5/6	Y	Explant	Y	N	Y
3	Radiculopathy	F	21	L4/5	N	Explant	Y	N	Y
4	Radiculopathy	F	47	C6/7	Y	Explant	Y	N	Y
5	Cauda equina syndrome	M	48	L5/S1	Y	Explant	Y	N	Y
6	Disc protrusion	F	44	L4/5	Y	Alginate	Y	N	Y
7	Sciatica	M	78	L4/5	Y	Alginate	Y	N	Y
8	Radiculopathy	M	45	C6/7	Y	Alginate	Y	N	Y
9	Trauma	F	55	L3/L4	N	Alginate	Y	N	N
10	Trauma	M	53	L1/L2	N	Alginate	Y	N	N
11	Trauma	M	31	L1/L2	N	Alginate	Y	N	N
12	Trauma	F	37	L1/L2	N	Alginate	Y	N	N
13	Trauma	F	38	L5/S1	N	Alginate	Y	N	Y
14	Trauma	M	60	L1/L2	N	Alginate	Y	N	Y
15	Trauma	M	37	Th12/L1	N	Alginate	Y	N	Y
16	—	F	65	L5/S1	Y	Paraffin embedding (NP/AF and CEP)	N	IL‐4, IL‐4R, IL‐10R	N
17	Disc prolapse	F	47	L5/S1	Y	Paraffin embedding (NP/AF and CEP)	N	IL‐4, IL‐4R, IL‐10R	N
18	Disc degeneration	M	70	C5/C6	Y	Paraffin embedding (NP/AF and CEP)	N	IL‐4, IL‐4R, IL‐10R	N
19	—	M	30	L5/S1	N	Paraffin embedding (NP/AF and CEP)	N	IL‐4, IL‐4R, IL‐10R	N
20	Recurrent Disc Prolapse	M	51	L4/L5	Y	Paraffin embedding (NP/AF)	N	IL‐4R, IL‐10R	N
21	Disc herniation	M	52	L2/L3	Y	Paraffin embedding (NP/AF and CEP)	N	IL‐4, IL‐4R, IL‐10R	N
22	Disc prolapse	F	35	L5/S1	Y	Paraffin Embedding (NP/AF and CEP)	N	IL‐4, IL‐10R	N
23	Recurrent Disc Prolapse	F	50	L5/S1	Y	Paraffin embedding (NP/AF and CEP)	N	IL‐4, IL‐4R, IL‐10R	N
24	Radicular Pain	F	—	L3/L4	N	Paraffin embedding (NP/AF and CEP)	N	IL‐4, IL‐4R, IL‐10R	N
25	Acute disc protrusion	M	42	L4/L5	N	Paraffin embedding (NP/AF and CEP)	N	IL‐4, IL‐4R, IL‐10R	N
26	Acute disc prolapse	F	38	L5/S1	Y	Paraffin embedding (NP/AF and CEP)	N	IL‐4, IL‐4R, IL‐10R	N
27	Stenosis	F	67	L4/L5	Y	Paraffin embedding (NP/AF and CEP)	N	IL‐4R, IL‐10R	N
28	Disc protrusion	F	28	L4/L5	Y	Paraffin embedding (NP/AF)	N	IL‐4, IL‐4R, IL‐10R	N
29	—	—	—	—	Y	Paraffin embedding (NP/AF and CEP)	N	IL‐4, IL‐4R, IL‐10R	N
30	—	M	57	C4/C7	N	Paraffin embedding (NP/AF and CEP)	N	IL‐4, IL‐10R	N
31	—	—	—	—	Y	Paraffin embedding (NP/AF and CEP)	N	IL‐4, IL‐4R, IL‐10R	N
32	Bilateral stenosis	M	21	L4/L5	Y	Paraffin embedding (NP/AF and CEP)	N	IL‐4, IL‐10R	N
33	—	—	—	—	Y	Paraffin embedding (NP/AF and CEP)	N	IL‐4, IL‐4R, IL‐10R	N
34	Cauda Equina syndrome	F	43	L5/S1	Y	Paraffin embedding (NP/AF and CEP)	N	IL‐4, IL‐4R	N
35	Disc prolapse	F	—	L5/S1	N	Paraffin embedding (NP/AF)	N	IL‐4, IL‐4R, IL‐10R	N
36	Impeding cauda equina	F	45	L5/S1	N	Paraffin embedding (NP/AF and CEP)	N	IL‐4, IL‐10R	N
37	Pseudarthrosis	F	56	L3/L4	Y	Paraffin embedding (NP/AF)	N	IL‐4R	N
*N*=					22 intact		14	22	11

Abbreviations: C, cervical; F, female; L, lumbar; M, male; N, no; Th, thoracic; Y, yes.

### 2.2 Alginate Beads Encapsulation

Isolated and expanded NP cells from trauma and degenerated discs were trypsinised, washed with PBS, and centrifuged at 500 g for 5 min. NP cells were then re‐suspended and homogenized with 1.2% w/v alginic acid (A2033, Sigma‐Aldrich) at a density of 4 × 10^6^ cells/ml, mimicking native NP cell density [20]. Encapsulation was performed by dropping the alginate mixture into 200 mM CaCl_2_ solution at constant speed through a 19 gauge needle. Alginate beads were washed twice in 0.15 M NaCl and low‐glucose (1 g/L) DMEM (LG‐DMEM) (Invitrogen) supplemented with 1% v/v ITS‐X (#51500–056, Invitrogen), 1% v/v P/S, 2.5 μg/mL Amphotericin B, 25 μg/mL L‐Ascorbic acid 2‐phosphate sesquimagnesium salt hydrate, 1% v/v L‐glutamine (25030–024, Invitrogen), 40 ug/ml L‐proline (#P5607, Sigma‐Aldrich), and 1,25 mg/mL Albumax (#11020–021, Invitrogen) (Complete NP media) [19] followed by 14 days of phenotype recovery culture under hypoxic conditions (5% O_2_) at 37°C before treatments. Medium was replaced three times per week. After the phenotype recovery period, alginate beads were either: (i) cultured for 2 days and subsequently treated with 10 ng/mL IL‐10 or IL‐4 (single treatments) or (ii) stimulated with 1 ng/mL IL‐1β for 2 days and subsequently treated with 10 ng/mL IL‐10 or IL‐4 (combined treatments). NP cells from trauma discs were cultured at physiological osmolarity (419.6 ± 1.2 mOsm/kg), while NP cells from degenerated discs were cultured at degenerative osmolarity (309.3 ± 6.1 mOsm/kg). Osmolality of the media was measured with an osmometer (OsmoTECH Single‐Sample Micro‐Osmometer, I&L Biosystems GmbH, Troisdorf, Germany).

### 2.3 Human NP Explant Extraction

Cores of human NP tissue (5 mm Ø and 6 mm high) from patients undergoing surgery were collected and placed into a ring culture system as described previously [21]. Explants were cultured under hypoxia conditions (5% O_2_) at 37°C with complete media for 5 days to allow recovery from isolation. Finally, explants were treated under the same stimulation groups as the NP cells from degenerative IVDs encapsulated in alginate beads.

### 2.4 Immunohistochemistry

IHC was used to determine local protein expression of IL‐4, IL‐10, IL4R, and IL10R within native human disc tissue. Briefly, 22 IVD tissues from human donors (Table 1) were immediately fixed in 10% (v/v) formalin before culture (Leica, Milton Kynes, UK) and embedded in paraffin wax. Four μm sections were mounted on positively charged slides and allowed to dry before immunohistochemical staining. IHC was performed using standard immunohistochemical procedures as previously reported [22] with specific details regarding, antigen retrieval methods, serum blockage, primary and secondary antibody details shown in Table 2. Digital IHC slide images were scanned using PANNORAMIC 250 Flash II DX (3DHistech, Budapest, Hungary) and visualized by SlideViewer (SlideViewer 2.8, 3DHistech). Image batch analysis for immunopositivity quantification was performed using Q‐Path analysis [23] (version 10.2.1 for Mac OS X) with a modified methodology for acellular tissues as reported previously [24].

**TABLE 2:** Target antibodies used for IHC with optimal concentrations and antigen retrieval methods.

Primary Ab	Clonality	Dilution	Antigen retrieval method	Secondary Ab	Blocking serum
IL‐4 (ab239508)	Mouse monoclonal	1:500 (2 μg/mL)	Non‐antigen retrieval	Rabbit (ab6727)	Rabbit serum
IL‐10 (ab134742)	Mouse monoclonal	1:200 (10 μg/mL)	Non‐antigen retrieval	Rabbit (ab6727)	Rabbit serum
IL‐4R (ab203398)	Rabbit monoclonal	1:200 (5 μg/mL)	Enzymatic	Goat (ab6720)	Goat serum
IL‐10 R (ab197666)	Rabbit polyclonal	1:250 (2.4 μg/mL)	Enzymatic	Goat (ab6720)	Goat serum

### 2.5 Quantitative Real‐Time (qRT)‐Polymerase Chain Reaction

Following treatments, NP cells in alginate beads and NP tissue explants were snap‐frozen in liquid nitrogen, pulverized using a precooled mortar, and transferred into 1 mL of Trizol (Life Technologies) for RNA extraction previously described [25]. Isolated RNA was transcribed to cDNA following the reverse transcription method applied to cow and human IVD samples previously described [19]. In the case of frozen alginate beads containing trauma NP cells, beads were pulverized and mixed with TRIzol reagent (#TR118, Molecular Research Center, Cincinnati, US, distributed by Lucerna‐Chem Inc., Switzerland) for downstream RNA extraction using Trizol‐silicon membrane purification [26]. RNA was reverse‐transcribed to cDNA utilizing a High‐Capacity cDNA Reverse Transcription kit (#4368814; Thermo‐Fisher Scientific, Basel, Switzerland) together with a MyCycler Thermal Cycler system (#1709703; Bio‐Rad Laboratories Inc., Cressier, Switzerland). Finally, the expression of genes of interest (Table S1) was determined by qRT‐PCR using either a StepOnePlus Real‐Time PCR System (Applied Biosystems, Warrington, UK) or a CFX96 Real‐Time System (#185–5096; Bio‐Rad Laboratories, Switzerland). Each sample was run in duplicate with 10 μL reaction volume, containing at least 10 ng cDNA using either predesigned primers (Applied Biosystems) and TaqMan FAST Universal PCR Master Mix (Applied Biosystems) on the QuantStudio 3 (Thermo‐Fisher Scientific) or iTaq Universal SYBR Green Supermix (#1725122; Bio‐Rad Laboratories) for a quantitative polymerase chain (qPCR) reaction on a CFX96 Real‐Time System (#185–5096; Bio‐Rad Laboratories) for 45 cycles. Relative gene expression was determined using the 2^−ΔΔCt^ method and normalized to the ribosomal 18S reference gene.

### 2.6 Luminex Assay

Conditioned media and protein lysates from IL‐4, IL‐1β single, and combined stimulations were collected and analyzed for 73 secreted proteins and 21 phosphorylated proteins using bead‐based Luminex multiplex immunoassays (Protavio, Athens, Greece) developed as previously described [27]. Six panels were used to measure 73 analytes in conditioned media using an eight‐point standard curve (Table S2), while two panels (Table S3) were used to measure phosphorylated proteins in protein lysates using positive (signal) and negative (noise) controls (Table S4). Ninety‐six‐well plates were coated with 50 μL of each 1× bead mix dilution (Mag‐Plex magnetic microspheres, Luminex Corp, Austin, TX, USA) (2500 beads per bead ID), and incubated with 35 μL of standards, samples, and blanks (Table S5) for 90 min at real‐time (RT) on an orbital shaker (1000 rpm). Wells were washed twice with assay buffer (PR‐ASSB‐1x, Protavio, Greece), and 20 μL of detection antibody mix at an average concentration of 1 μg/mL was added to each well and incubated for 60 min at RT on an orbital shaker at 1000 rpm. After washing twice with assay buffer, 35 μL of Streptavidin, R‐Phycoeythrin conjugate at a concentration of 5 μg/mL (SAPE‐001, MOSS, USA) was added to each well and incubated for 15 min at RT on an orbital shaker at 1000 rpm. Finally, wells were washed twice, resuspended in 130 μL assay buffer, and measured in Luminex 3D (Luminex FLEXMAP 3D platform, Luminex Corp., Austin, TX, USA) using a minimum of 100 counts to determine median fluorescence intensity values.

### 2.7 Statistical Analysis

All quantitative data were assumed to follow a non‐parametric distribution. Statistical analyses for gene expression, secretome quantification, and IHC data were performed using a Kruskal–Wallis test and Dunn's multiple comparisons *post hoc* test. In addition, the Mann–Whitney rank‐sum statistical test was performed for IL‐4 or IL‐10–treated secretome profile comparison. All statistical analyses were performed with GraphPad Prism (version 10.2.1 for Mac OS X, GraphPad Software; San Diego, CA, USA) and R (R Core Team, 2020), RStudio (R version 4.4.1, R studio Team, 2020) software including rstatix [28] and stats [29] packages and a *p*‐value < 0.05 was considered statistically significant. All quantitative results are presented as median and the exact number of biological (N) and technical (n) replicates are indicated in the respective figure legend.

## 3 Results

### 3.1 IL‐4, IL‐4R, IL‐10, and IL‐10R Are Expressed in Human IVD Tissue

**FIGURE 1:**
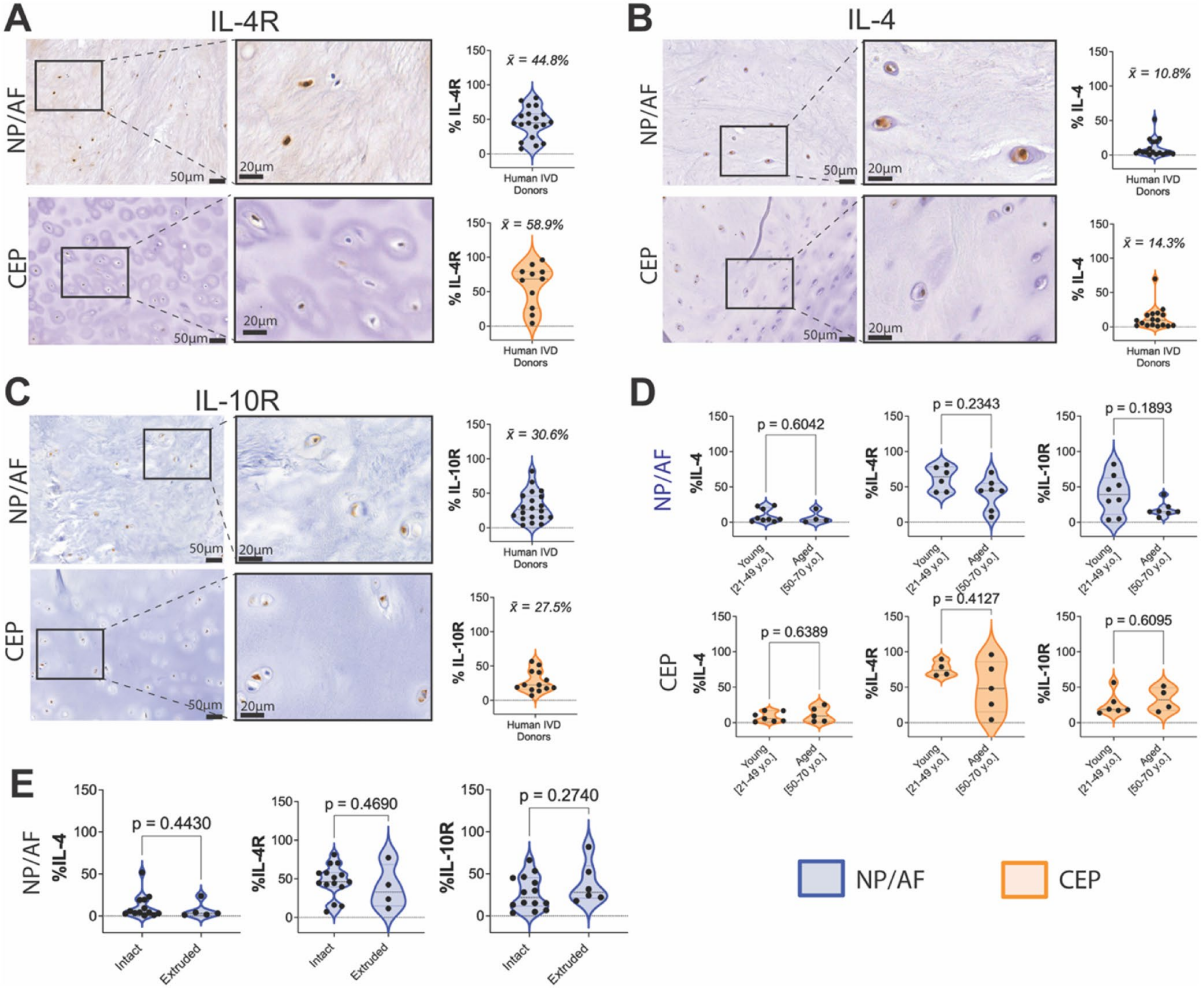
Immunohistochemical staining and immunopositivity percentages of (A) IL‐4R, (B) IL‐4, and (C) IL‐10R in human NP/AF and CEP tissue. (D) IL‐4, IL‐4R, and IL‐10R immunopositivity rates on human NP/AF and CEP according to donor age. (E) L‐4, IL‐4R, and IL‐10R immunopositivity rates on human NP/AF tissue with or without NP tissue extrusion. Kruskal–Wallis test and a Dunn's multiple comparisons post hoc test rank‐sum statistical test were performed with a *p‐*value < 0.05 which was considered statistically significant (*) (*N* = 11–29). Objective magnification = 20× and 40×; scale bar = 50 μm and 20 μm. Blue = NP/AF; Orange = CEP.

IL‐4, IL‐4R, and IL‐10R immunopositivity was observed in native human NP/AF tissue (Figure 1). The percentage of IL‐4R, IL‐4, and IL‐10 immunopositivity was quantified in human NP/AF and CEP tissue samples (Table 1) obtaining 44.8% IL‐4R, 10.8% IL‐4, 30.6% IL‐10R in NP/AF, 58.9% IL‐4R, 14.3% IL‐4, and 27.5% IL‐10R in CEP regions (Figure 1A–C). There were no significant differences (*p* > 0.05) in IL‐4, IL‐4R, and IL‐10R immunopositivity between young and aged donors. Nevertheless, a decrease of 21.9% and 21.5% immunopositivity for IL‐4R and IL‐10R was seen in NP/AF tissue from aged v/s young donors (Figure 1D). Furthermore, no difference in IL‐4, IL‐4R, and IL‐10R was seen between intact and extruded discs (*p* > 0.05) (Figure 1E).

### 3.2 IL‐1β Enhances Catabolic Gene Expression Only in Primary Trauma NP Cells

**FIGURE 2:**
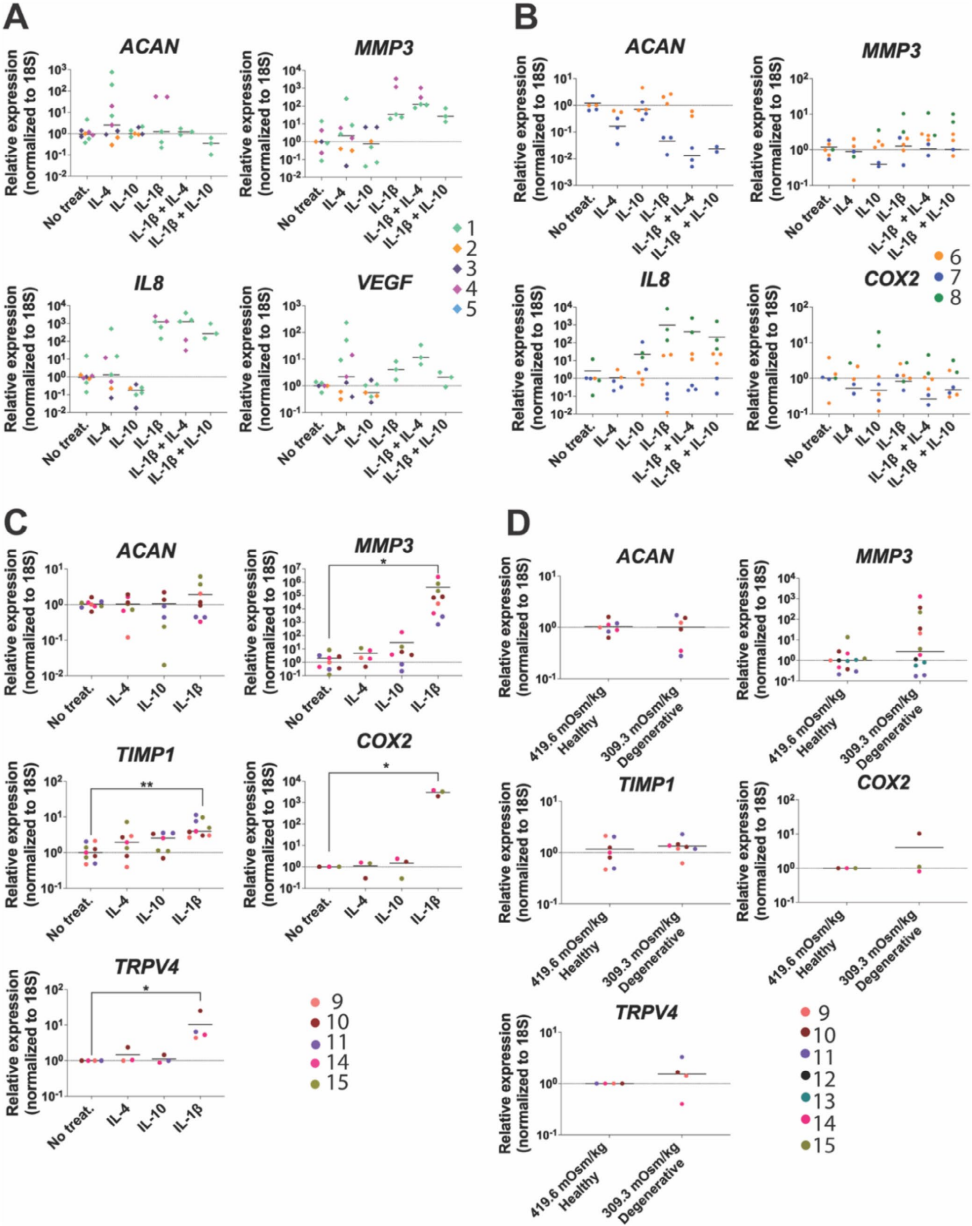
Relative gene expression of *ACAN*, *MMP3*, IL8, *TIMP1*, *COX2*, VEGF, and TRPV4 in primary human (A) explants, (B) degenerative, and (C) trauma cells. (D) Relative gene expression of *MMP3*, *COX2*, *TIMP1*, *ACAN*, and TRPV4 in trauma NP cells under degenerative (309.3 ± 6.1 mOsm/kg) and healthy (419.6 ± 1.2 mOsm/kg) osmolarities. Each replicate with each patient is defined as a separate color, the median value for treatment or osmolarity condition is shown (*N* = 3–5, *n* = 1–3). Kruskal–Wallis test and a Dunn's multiple comparisons post hoc test rank‐sum statistical test were performed with a *p*‐value < 0.05 which was considered statistically significant (*).

No significant differences were observed between IL‐4 and IL‐10 single or combined treatments on gene expression of catabolic markers *MMP3, IL‐8*, or vascular endothelial growth factor (VEGF) as well as on anabolic matrix protein: Aggrecan (ACAN) in NP explants (Figure 2A). IL‐1β–containing groups exhibited upregulated *MMP3*, *IL‐8*, and VEGF gene expression (Figure 2A). No significant effects on gene expression of *MMP3*, Cyclooxygenase 2b (*COX2*), *IL‐8*, and *ACAN* were observed following stimulation with IL‐4, IL‐10, or IL‐1β in human NP cells from degenerate discs (Figure 2B). In contrast, human NP cells from trauma discs showed significant upregulation of *MMP3 (p < 0.05), COX2 (p < 0.05), TIMP1* (*p* < 0.01), and TRPV4 (*p* < 0.05) gene expression following IL‐1β stimulation (Figure 2C). No significant differences were observed between degenerative and healthy osmolarity conditions in NP cells from trauma discs (Figure 2D).

### 3.3 Ex Vivo and In Vitro Human NP Cell's Secretomes Differ Under IL‐4 and IL‐10 Stimulation

**FIGURE 3:**
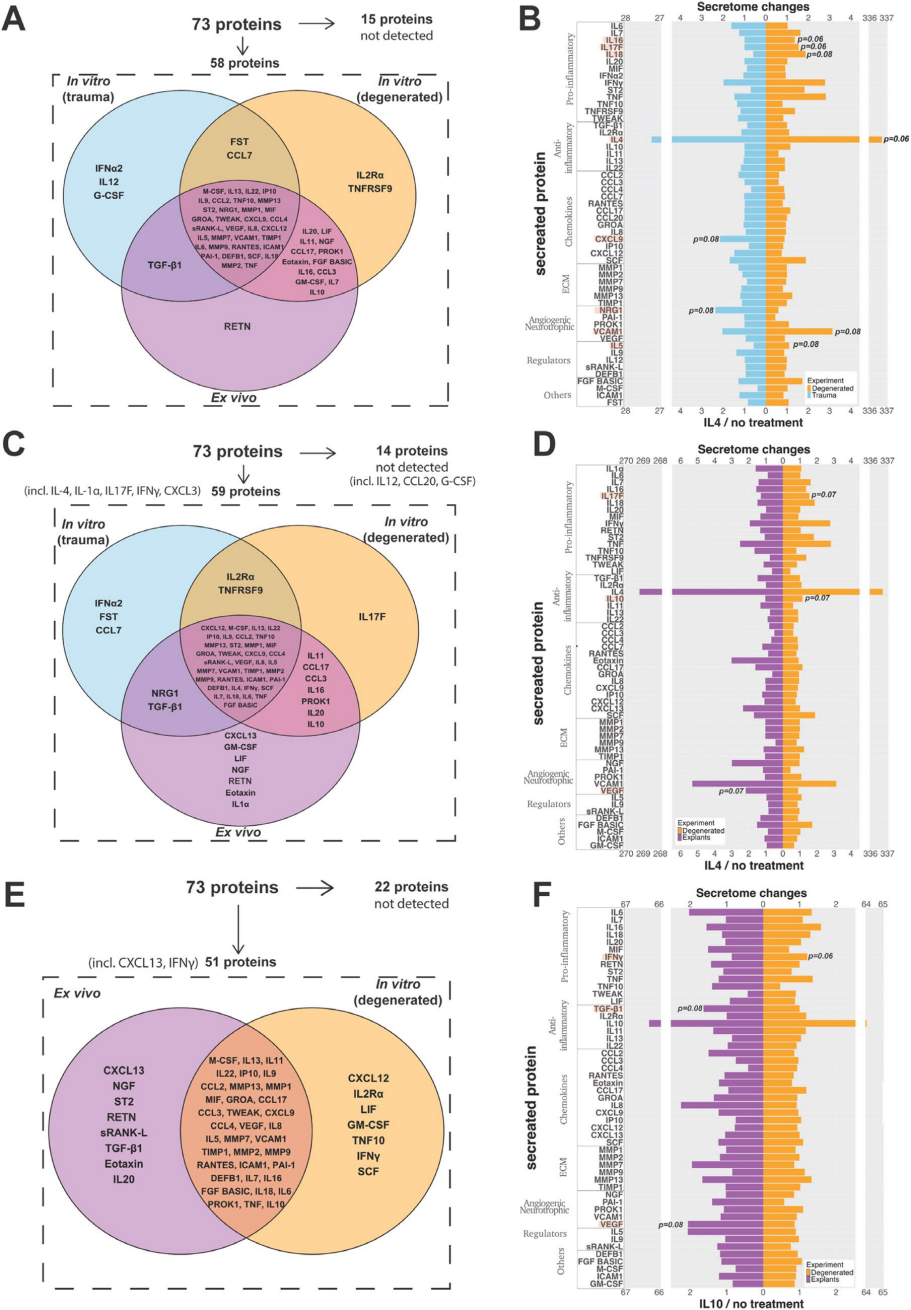
Baseline, IL‐4, and IL‐10 stimulated protein secretion profiling from primary human trauma cells, primary human degenerated cells, and human explant cultures. Venn's diagram shows in vitro trauma, degenerated NP cells, and ex vivo NP explant (A) baseline, (B) IL‐4 stimulated, and (C) IL‐10 stimulated secretome profiles. Divergent bar chart comparing IL4 effect on (D) trauma and degenerated NP cell secretome, (E) explants and degenerated NP cell secretome. (F) Divergent bar chart comparing IL‐10 effect on explants and degenerated NP cell secretome. Each bar represents one secreted protein normalized to no‐treatment control from human trauma or degenerated IVDs (*N* = 3–5, *n* = 1–3) and the Mann–Whitney rank‐sum statistical test was performed with a *p*‐value < 0.05 which was considered statistically significant and *p*‐value < 0.08 close to significant (in red).

Each culture system exhibited unique secreted protein patterns indicating a possible influence of the culture system on the secretome profile (Figure 3A,C,E). Interestingly, IL‐4 stimulated NP cells and explants showed an enhanced catabolic environment, where IL‐1, IL‐17F, CXCL13, and IFNγ were detected in contrast to the no‐treatment baseline (Figure 3A,C). Similarly, IL‐10 treatment also promoted CXCL13 and IFNγ secretion in NP explants and cells, respectively (Figure 3E). IL‐4 treatment showed differential responses in NP cells isolated from trauma or degenerated discs, with non‐significant increases of IL‐5 (*p* = 0.08), IL‐18 (*p* = 0.08), IL‐17F (*p* = 0.06), IL‐16 (*p* = 0.06), vascular cell adhesion 1 (*p* = 0.08), and IL‐4 (*p* = 0.06) protein secretion in NP cells from degenerated discs. NP cells from trauma discs showed non‐significant increases in protein secretion of CXCL9 (*p* = 0.08), and neuregulin 1 (NRG1) (*p* = 0.08) (Figure 3B). In addition, NP cells and explants from degenerated discs shared a similar secretome profile following IL‐4 treatment despite a non‐significant increase in IL‐17F (*p* = 0.07) and IL‐10 (*p* = 0.07) and non‐significant decrease in VEGF (*p* = 0.07) protein secretion in cell cultures (Figure 3D). IL‐10–treated NP cells and explants from degenerated discs also shared a similar pattern with only non‐significant increases in VEGF (*p* = 0.08) and TGFβ1 (*p* = 0.08) and a decrease in IFNγ secretion (*p* = 0.06) in explants (Figure 3F).

### 3.4 IL‐1β Promotes Catabolism in Primary Trauma NP Cells at the Secretome Level

**FIGURE 4:**
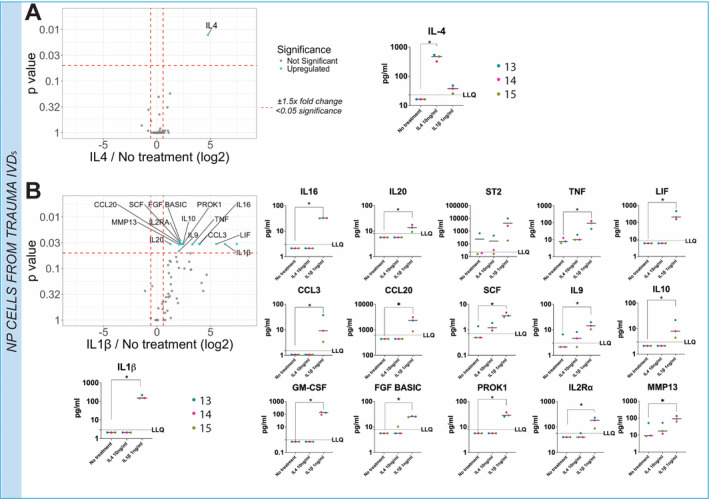
Primary NP cell from trauma patients secretome changes under 10 ng/mL IL‐4 or 1 ng/mL IL‐1β stimulation. Volcano plot comparing (A) IL‐4 secretome profile to no‐treatment. (B) Volcano plot comparing IL‐1β secretome profile to no‐treatment. The log2 fold change indicates the expression of human NP trauma cell‐secreted factors compared with no‐treatment control. Each dot represents one secreted protein. Each replicate with each patient is defined as a separate color, the median value for treatment is shown (*N* = 3, *n* = 1). Kruskal–Wallis test and a Dunn's multiple comparisons post hoc test were performed with a *p*‐value < 0.05 which was considered statistically significant (*).

Secretome from 10 ng/mL IL‐4 and 1 ng/mL IL‐1β stimulation in human trauma NP cells was compared with the no‐treatment condition. No significant changes were observed between IL‐4 stimulated and no‐treatment secretome patterns except for significantly upregulated IL‐4 protein expression, which was exogenously added as a treatment (Figure 4A). In contrast, pro‐inflammatory cytokine and chemokine secretion, including IL‐16, IL‐20, ST2, TNF, leukemia inhibitor factor, CCL3, CCL20, and stem cell factor (SCF), were significantly increased under IL‐1β stimulation alongside IL‐1β protein which was exogenously added as a treatment (*p* < 0.05) (Figure 4B). Similarly, regulators and adhesion factors such as IL‐9, granulocyte‐myeloid colony stimulation factor, basic fibroblast growth factor 2 (bFGF2), and Prokineticin 1 (PROK‐1) secretion were significantly increased by IL‐1β stimulation (*p* < 0.05) (Figure 4B). Anti‐inflammatory IL‐2Rα and IL‐10 protein secretion, and MMP13 secretion, were also significantly upregulated by IL‐1β (*p* < 0.05, Figure 4B).

### 3.5 IL‐1β Treatment Increases NF‐κB and c‐JUN Phosphorylated Forms in Trauma NP Cells

**FIGURE 5:**
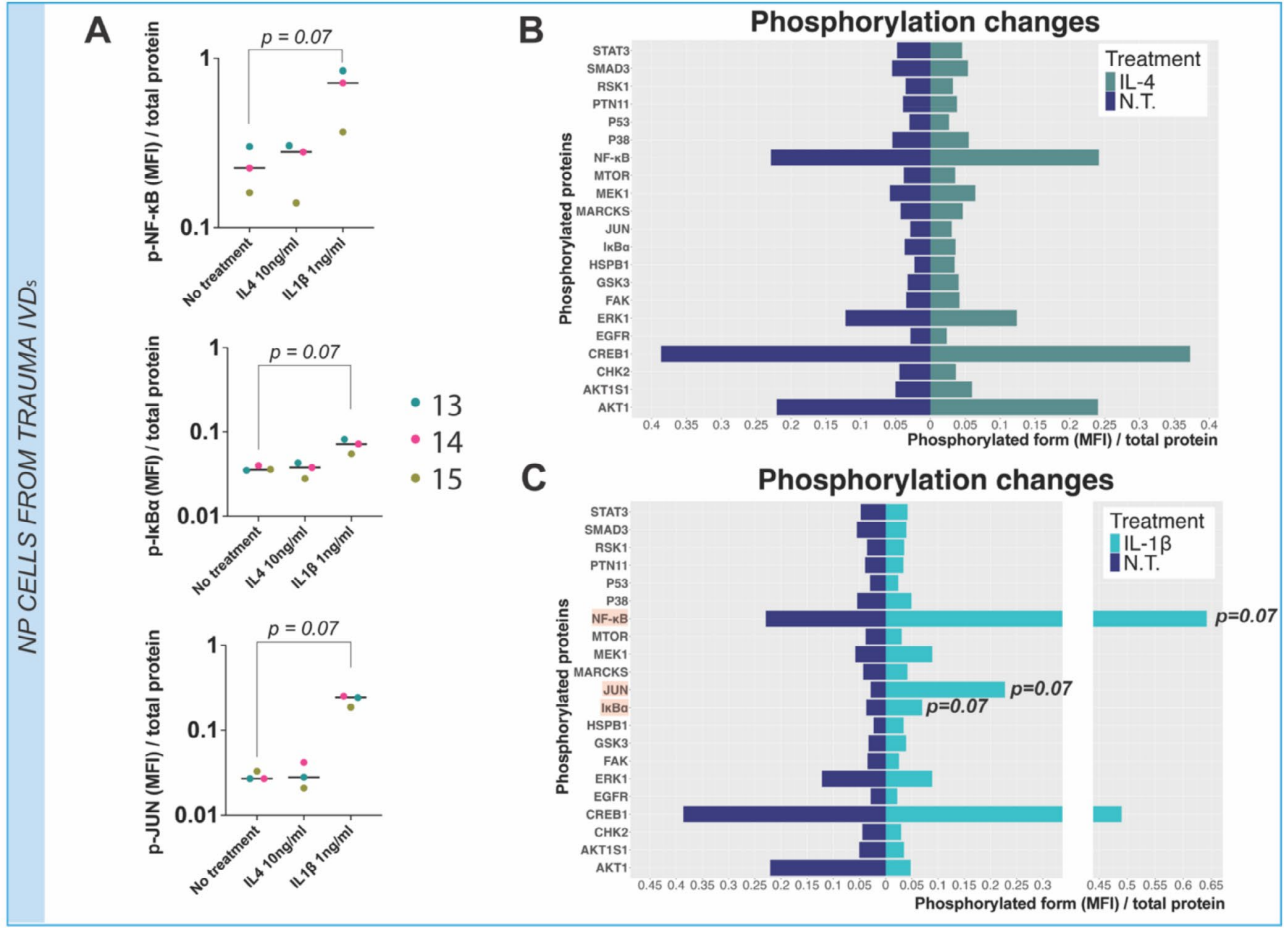
IL4 and IL‐1β induced phospho‐proteome profiles on primary NP cells from trauma patients. Each bar/dot represents one phosphorylated protein normalized to the total protein amount. Each patient is represented in a separate color, and the median value for treatment is shown (*N* = 3, *n* = 1). Kruskal–Wallis test and a Dunn's multiple comparisons post hoc test were performed with a *p*‐value < 0.05 which was considered statistically significant. MFI = mean fluorescence intensity.

To further analyze the downstream response of IL‐1β treatment and confirm the lack of effect of IL‐4 treatment on NP cells from trauma discs, key protein phosphorylation patterns related to inflammation/catabolism (NF‐κB, JUN, STAT3, MAPK/ERK, and MARCKS pathways), cell growth and survival (p53, mTOR/AKT, and GSK3 pathways), and mechanotransduction/adhesion (FAK pathway) were examined. Similar phosphorylation profiles were observed between untreated and IL‐4 stimulated NP cells, with no significant differences on phosphorylation levels normalized to total protein amount (Figure 5A,B). IL‐1β stimulation induced non‐significant increases of pNF‐κB (*p* = 0.07), pIκBα (*p* = 0.07), and pJUN (*p* = 0.07) (Figure 5A,C).

### 3.6 IL‐4 Enhances Catabolic Effect of IL‐1β in Human NP Degenerated Cells

**FIGURE 6:**
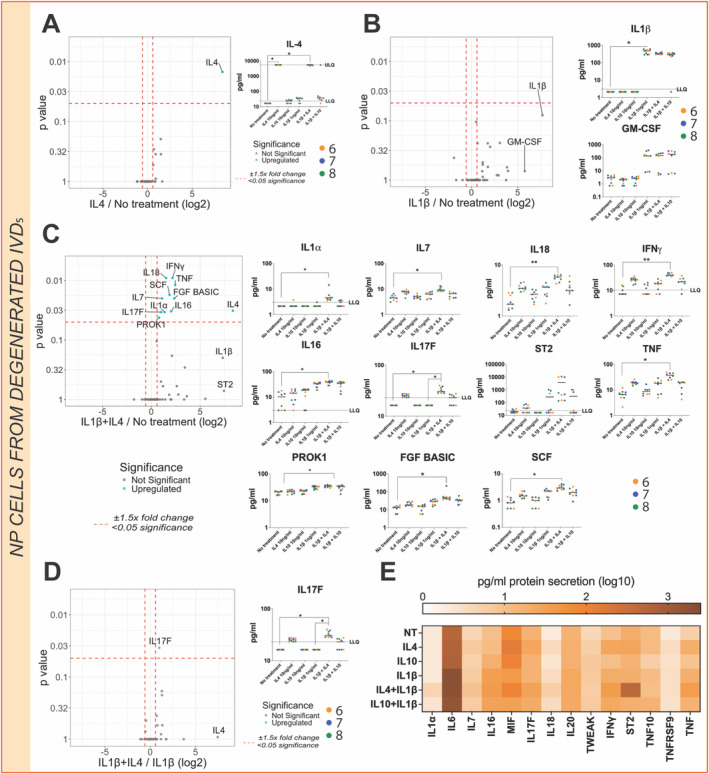
Primary NP cells secretome from degenerated IVDs under 10 ng/mL IL‐4, 1 ng/mL IL‐1β, or IL‐1β and IL‐4 combined stimulation. (A) Volcano plot comparing IL‐4 secretome profile to no‐treatment. (B) Volcano plot comparing IL‐1β secretome profile to no‐treatment. (C) Volcano plot comparing IL‐1β and IL‐4 combined secretome profile to no‐treatment. (D) Volcano plot comparing IL‐1β and IL‐4 combined secretome profile to IL‐1β baseline. The log2 fold change indicates the expression of human NP degenerated cell‐secreted factors. Each dot represents one secreted protein. Each replicate with each patient is defined as a separate color, the median value for treatment is shown (*N* = 3, *n* = 3). Kruskal–Wallis test and a Dunn's multiple comparisons post hoc test were performed with a *p*‐value < 0.05 which was considered statistically significant (*). (E) Heat map of pro‐inflammatory secreted protein levels under single 10 ng/mL IL‐4, 1 ng/mL IL‐1β, or IL‐4 and IL‐1β combined stimulation. Each box corresponds to three donors and three technical replicate means values. The color indicates a log10 fold‐change.

In contrast to the NP cell secretome profile from trauma IVDs, NP cells from degenerated IVDs showed a different response pattern to IL‐4 and IL‐1β treatment. No significant differences in the secretome following single treatments of IL‐4 or IL‐1β were observed (Figure 6A,B). However when combined IL‐4 and IL‐1β stimulation induced a catabolic effect inducing a significant increase of pro‐inflammatory protein secretion: IL‐1α (*p* < 0.05), IL‐7 (*p* < 0.05), IL‐16 (*p* < 0.05), IL‐17F (*p* < 0.05), IL‐18 (*p* < 0.01), IFNγ (*p* < 0.01), TNF (*p* < 0.05), ST2 (p < 0.05) as well as PROK1, bFGF2, and SCF (*p* < 0.05) compared with unstimulated cells (Figure 6C,E). Moreover, IL‐17F protein secretion was also significantly increased (*p* > 0.05) under IL‐4 and IL‐1β combined treatment compared with protein secretion from cells only treated with IL‐1β, highlighting IL‐4's catabolic effect (Figure 6D).

### 3.7 IL‐10 Possesses a Less Effective Response in Human NP‐Degenerated Cells

**FIGURE 7:**
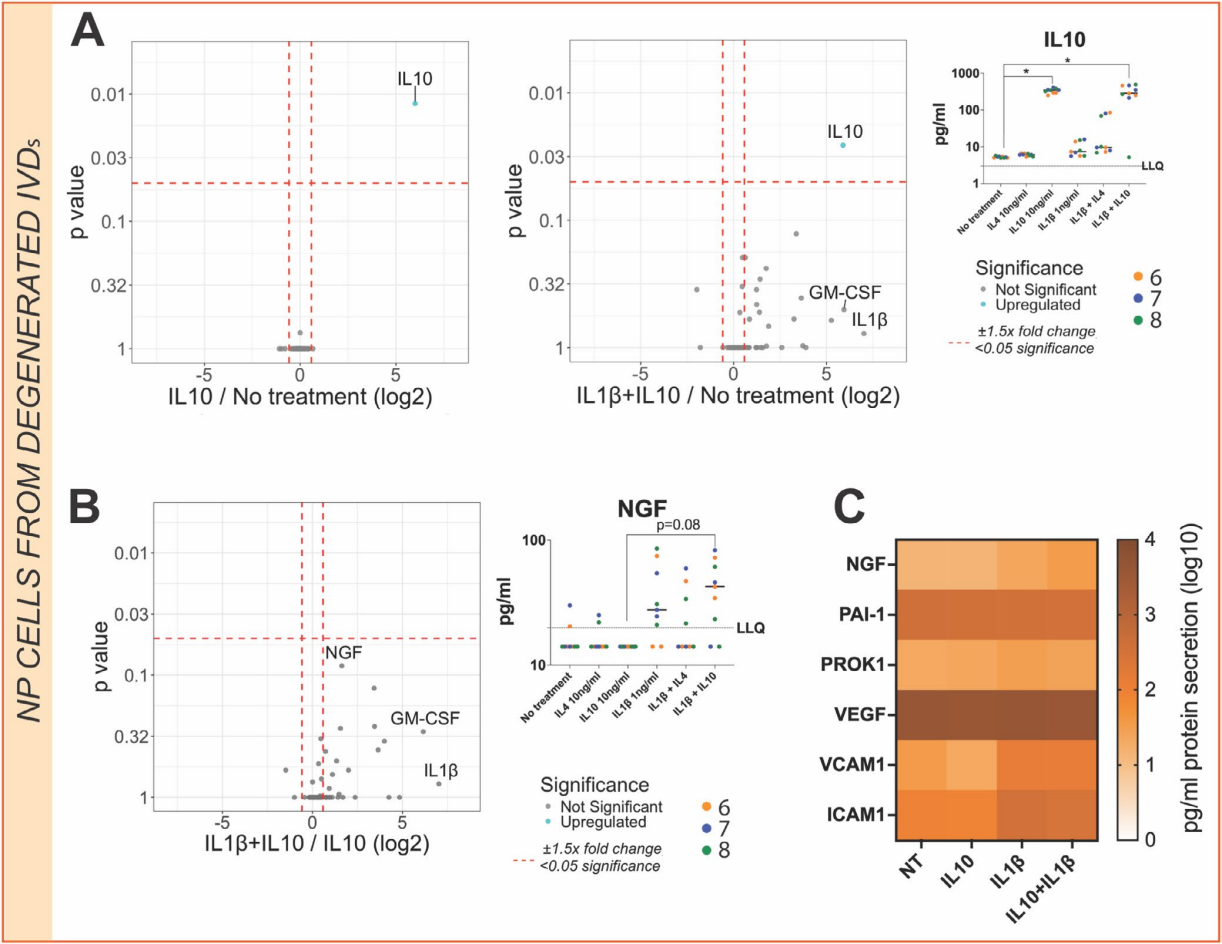
Primary NP cells from degenerated IVDs secretome changes under single 10 ng/mL IL‐10, 1 ng/mL IL‐1β, or combined IL‐10 and IL‐1β stimulation. (A) Volcano plot comparing IL‐10 secretome profile to no‐treatment. (B) Volcano plot comparing IL‐1β secretome profile to no‐treatment. The log2 fold change indicates expression of human NP degenerated cell secreted factors. Each dot represents one secreted protein. Each replicate with each patient is represented as a separate color, the median value for treatment is shown (*N* = 3, *n* = 3). Kruskal–Wallis test and a Dunn's multiple comparisons post hoc test was performed with a *p*‐value < 0.05 which was considered statistically significant. (C) Heat map of added treatments and neurotrophic and angiogenic secreted protein levels under single 10 ng/mL IL‐10, 1 ng/mL IL‐1β or IL‐10, and IL‐1β combined stimulation. Each box corresponds to three donors and three technical replicates mean value. The color indicates log10 fold‐change.

The role of IL‐10 was also investigated at the secretome level in primary human NP cells from degenerated discs. No significant differences in the secretome were observed between no‐treatment and IL‐10 treatment or IL‐10 and IL‐1β combined treatment except for IL‐10 secretion, which was exogenously added as a treatment (Figure 7A). Interestingly, neurotrophic growth factor (NGF) secretion was slightly increased under IL‐10 and IL‐1β combined treatment, although this failed to reach significance (*p* = 0.08) compared with the IL‐10 baseline (Figure 7B,C).

### 3.8 IL‐4 and IL‐10 Stimulation in Human NP Explants Might Impact Chemokine Secretion

**FIGURE 8:**
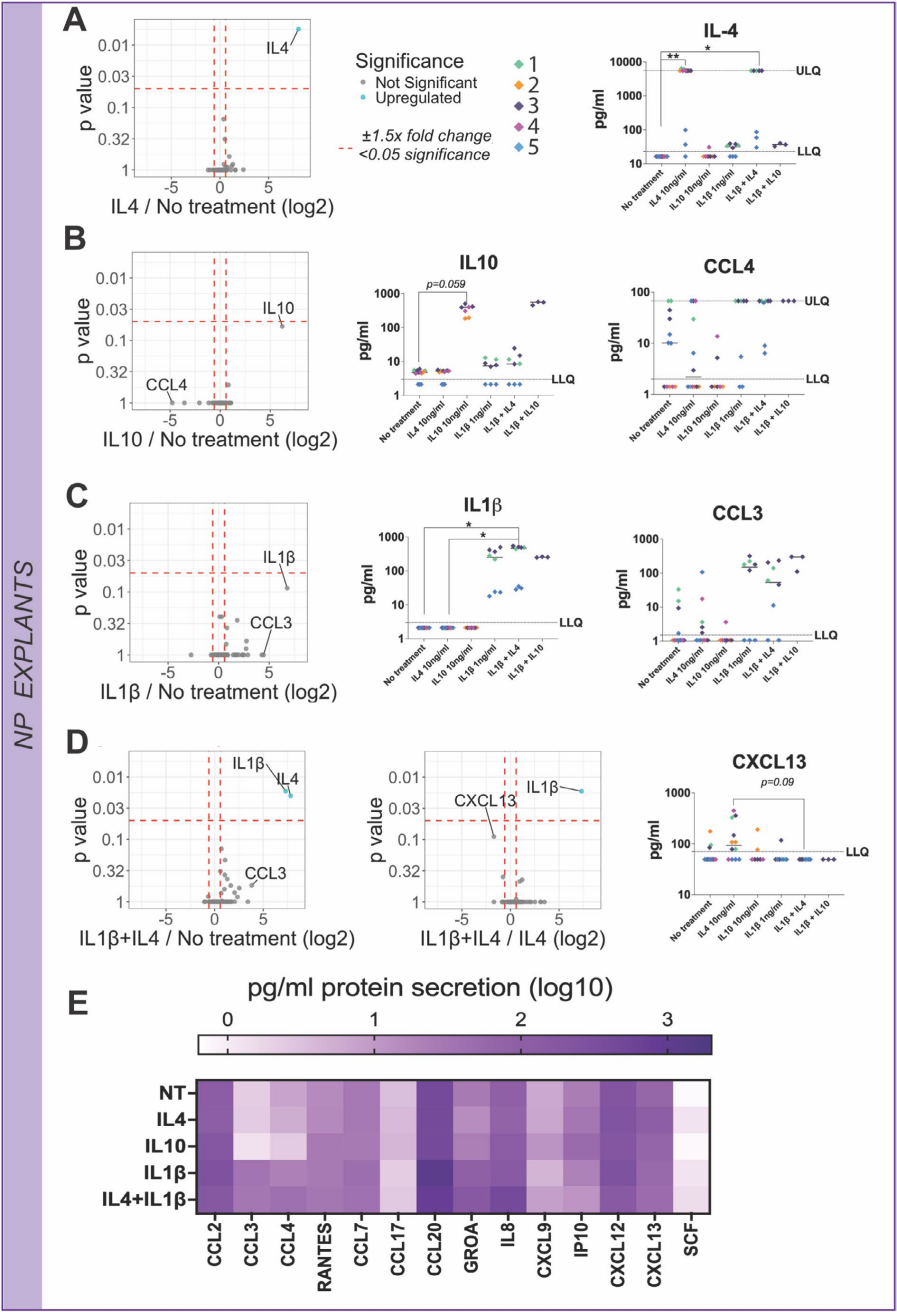
10 ng/mL IL‐4, IL‐10, and 1 ng/mL IL‐1β stimulated human NP explants secretome changes. (A) Volcano plot comparing IL‐4 secretome profile to no‐treatment. (B) Volcano plot comparing IL‐10 secretome profile to no‐treatment. (C) Volcano plot comparing IL‐1β secretome profile to no‐treatment. (D) Volcano plots comparing IL‐4 and IL‐1β combined treatment secretome profile to no‐treatment and IL‐4 baseline. The log2 fold change indicates the expression of human NP explants' secreted factors. Each dot represents one secreted protein. Each replicate with each patient represented a separate color, the median value for treatment is shown (*N* = 3–5, *n* = 3). Kruskal–Wallis test and a Dunn's multiple comparisons post hoc test were performed with a *p*‐value < 0.05; which was considered statistically significant. (E) Heat map of secreted chemokine protein levels under single 10 ng/mL IL‐10, 10 ng/mL IL‐4, 1 ng/mL IL‐1β, or IL‐1β combined stimulation. Each box corresponds to 3–5 donors, and three technical replicates mean value. The color indicates log10 fold‐change.

Next, the response of NP tissue explants was determined. No significant differences were observed for IL‐4 or IL‐10 treatments compared with no‐treatment except for increased IL‐4 and IL‐10 protein secretion, respectively, exogenously added as a treatment in each case (Figure 8A,B). Interestingly, median CCL4 secretion was slightly decreased under IL‐10 treatment despite high variability between donors (Figure 8B). Conversely, CCL3 was increased in two out of three donors under IL‐1β treatment (Figure 8C). In the IL‐4 and IL‐1β combined treatment, CXCL13 secretion was slightly decreased compared with CXCL13 secretion in the IL‐4 treated group; however, this failed to reach significance (*p* = 0.09) (Figure 8D). IL‐4 and IL‐1β combined treatment exhibited a similar secretome pattern compared with IL‐1β single treatment, except for slightly increased IL‐8 and decreased CCL20 secretion (Figure 8E).

## 4 Discussion

IL‐4 and IL‐10 are well‐known immunomodulatory cytokines that act as anti‐inflammatory mediators in degenerative joint diseases, including rheumatoid arthritis [30] and osteoarthritis [31]. Nevertheless, their presence and role in IVD degeneration remains obscure. This study demonstrates for the first time the presence and localization of IL‐4, IL‐4R, and IL‐10R in human CEP and IL‐10R in human NP and AF tissue. IL‐4 cytokine and IL‐4R presence was also confirmed at the tissue level in human NP/AF region as previously reported [32, 33]. Interestingly, IL‐4R and IL‐10R immunopositivity was higher compared with IL‐4 immunopositivity in both NP/AF and CEP regions, similar to previously reported IL‐1 and IL‐1R expression patterns on human non‐degenerated and degenerated NP and inner AF samples [34]. Furthermore, extruded NP tissue samples revealed no significant changes in immunopositivity rates. Thus, we demonstrate that IL‐4R, IL‐10R, and IL‐4 are expressed by human native NP, AF, and CEP cells, and that NP extrusion does not influence their expression.

To further elucidate NP tissue and cell responses to IL‐4 and IL‐10 cytokines, mRNA expression of catabolic, anabolic, inflammatory, angiogenic, and mechanotransduction markers were investigated. No significant differences in gene expression were observed following 2 days of either 10 ng/mL IL‐4 or IL‐10 stimulation in any culture model (degenerated/non‐degenerated cells, degenerated explants). However, the lack of cellular response to these cytokines might be due to the relatively long treatment period, since IL‐4 downstream cascade activation through the well‐known STAT6 phosphorylation pathway [35] has been observed in IVD cells after 30 min of treatment [16]. In addition, IL‐4–dependent gene expression changes on IVD cells have been previously reported after 24 h of treatment [16]. Interestingly, NP cells from trauma and degenerated discs showed different gene expression responses to 1 ng/mL IL‐1β treatment. Specifically, NP cells from trauma patients exhibited a significant increase of catabolic, inflammatory, and mechanotransduction mediators under IL‐1β stimulation, similar to previous studies. In contrast, no significant effects on gene expression were observed in NP cells from degenerative IVDs, possibly due to an enhanced IL‐1β presence and induced catabolic baseline typically present in degenerated IVDs. In addition, increased osmotic environments have been previously reported to impact mRNA expression in human and bovine IVD cultures, the influence of osmolarity on gene expression was also evaluated. However, when exposing NP cells from trauma IVDs to both degenerative and healthy osmolarities, no significant differences were observed in catabolic, anabolic, inflammatory, angiogenic, or mechanotransduction gene expression, in contrast to prior osmolarity studies [36, 37].

In vitro treatment with IL‐4, IL‐10, and/or IL‐1β was performed to characterize the secretome profiles of NP cells from trauma and degenerated IVDs. Secretome patterns were markedly different between trauma and degenerated cell subsets after IL‐4 stimulation, suggesting that IL‐4 has a pleiotropic effect which depends on the degree of degeneration. Specifically, IL‐4 treatment exhibited a redundant effect on the NP cell's secretome and downstream signaling pathways from trauma patients. In contrast, IL‐1β stimulation promoted an expected catabolic response upregulating pro‐inflammatory markers and chemokine secretion as well as activating inflammation‐related signaling pathways as previously reported [38, 39]. However, IL‐1β treatment on NP cells from degenerated IVDs was not able to induce a catabolic response. Notably, IL‐1β protein level baselines were measured in both trauma and degenerated NP cells encapsulated in alginate and were found to be under the limit of quantification (3.0 pg/mL) in both cases. Interestingly, IL‐4 treatment combined with IL‐1β treatment on NP cells from degenerated IVDs exhibited a strong catabolic response at the secretome level. In particular, the presence of IL‐4 promoted a significant upregulation of the pro‐inflammatory marker IL‐17F, previously linked with IL‐4 and IL‐13 co‐activation of IL‐4 receptor type 2 [40]. Thus, IL‐4 could boost the catabolic effect of IL‐1β exclusively on NP cells from degenerative IVDs. In contrast, anti‐inflammatory properties [16] have been formerly attributed to IL‐4 cytokine on IVD cells and analgesic, chondroprotective activity in combination with IL‐10 in osteoarthritis [31, 41] and rheumatoid arthritis [30]. Nevertheless, previous studies on IL‐4–treated IVD cells might have neglected its pro‐inflammatory effect due to the absence of a 3D environment or physiological conditions (i.e., hypoxia). Taken together, the pleiotropic effect of IL‐4 could be due to degree of degeneration and environmental conditions. No significant effects were observed following IL‐10 treatment alone in vitro, while in the presence of IL‐1β, NGF secretion was slightly upregulated. Prior studies have reported IL‐1β induction of factors which drive innervation in IVD cells [42], which is associated with LBP [43]. Similarly, elevated IL‐10 levels have been identified in patients with LBP [44]. Taken together, a possible link between the effect of IL‐10 on neurotrophic factors and LBP might occur in the presence of IL‐1 catabolic stimuli.

In line with our study, ex vivo IL‐4, IL‐10, and IL‐1β stimulations were also performed to further investigate the impact of different culture systems on the pleiotropic effect of IL‐4 and IL‐10. No secretome changes were observed in NP explants stimulated with single or combined IL‐4 treatments, in contrast to former in vitro observations. Nevertheless, CXCL13 chemokine secretion was decreased under combined IL‐4 and IL‐1β treatment compared with the IL‐4 baseline, suggesting possible IL‐4 derived immunomodulation of IL‐1β through reduction of CXCL13 secretion. Interestingly, CXCL13 has been previously associated as a gene biomarker for LBP [45]. Furthermore, upregulated CXCL13 has been found in spinal cord astrocytes after spinal cord nerve ligation in a rodent model. At the same time, intrathecal injection of CXCL13 has also been reported as a possible cause of hyperalgesia [46]. Curiously, IL‐4 and IL‐10 fusion protein treatment inhibits dose‐dependent TNF secretion in primary spinal cord microglia [41], suggesting a potential interplay between IL‐4 and pain that might arise due to CXCL13 upregulation. Nevertheless, single IL‐10 or IL‐1β treatments on NP explants exhibited chemokine modulation, slightly decreasing CCL3 and increasing CCL4 secretion, respectively. Thus, the role of IL‐4 and IL‐10 in NP explants might be associated with IL‐1β ability for chemokine induction previously observed on human NP tissue. IL‐4 presence might modulate the effect of IL‐1β, as previously mentioned, on CXCL13 chemokine secretion.

Based on the presented results, we conclude that the phenotype of NP cells, i.e., trauma or degenerated, is crucial to determine the pleiotropic effect of IL‐4, promoting pro‐inflammatory responses in the presence of catabolic stimuli as well as enhancing the catabolic response of IL‐1β in NP cells from degenerated IVDs. Thus, our initial hypothesis stating the anabolic nature of IL‐4 was rejected due to a lack of anabolic response under IL‐4 stimulation. Furthermore, IL‐4 treatments combined with IL‐1β promoted pro‐inflammatory response, differing from the hypothesized anabolic rescue. Similarly, environmental conditions such as a 3D environment or hypoxia might influence the role of IL‐4 in IVDs, potentially shifting its anti‐inflammatory action toward a pro‐inflammatory scenario. Nevertheless, IL‐4 treatment and IL‐1β cytokine exhibited different roles in decreasing CXCL13, suggesting a possible association with pain. In addition, the role of IL‐10 remains unclear, although it might be linked to neurotrophic properties. Therefore, the hypothesized anabolic effect of IL‐10 remains obscure. Overall, further investigations on the pleiotropic effects of IL‐4 and IL‐10 are required to fully understand their anti or pro‐inflammatory nature during IVD degeneration.

## Author Contributions

P.B.‐L., S.T., B.G., C.L.M., and J.N. performed the conceptualization. P.B.‐L. ran the main experiments, wrote the main text, performed the data analysis, and prepared the visualizations and graphs. S.T. contributed to writing and running experiments. P.B.‐L. and A.N. optimized ab staining, A.N. performed ab batch staining. E.K., S.T., and K.B.C. executed Luminex measurements. K.B.C., K.W.‐K., C.L.M., L.G.A., B.G. contributed with writing and reviewing. B.G., C.L.M., K.W.‐K., L.G.A., and J.N. reviewed and edited the manuscript and sourced funding. All authors approved the final version of the manuscript.

## Conflicts of Interest

E.K. and L.G.A. were employed by Protavio Ltd. All other authors do not have any conflicts of interest to report. The remaining authors declare that the research was conducted in the absence of any commercial or financial relationships that could be construed as a potential conflict of interest.

## Supporting information


Table S1.

Table S2.

Table S3.

Table S4.

Table S5.
