# Supplementary material for: Ex Vivo and In Vitro Proteomic Approach to Elucidate the Relevance of IL‐4 and IL‐10 in Intervertebral Disc Pathophysiology
Source: JOR Spine. 2025 Feb 10;8(1):e70048. doi: 10.1002/jsp2.70048 (PMC11808320; doi:10.1002/jsp2.70048)
Supplement: Supplementary file 1 — Table S1. [file JSP2-8-e70048-s003.docx]

**Table S1**: Investigated gene overview, qRT-PCR method and primers used in this study.

| **Gene type** | **Symbol** | **qRT-PCR method** | **Forward and Reverse**    **Primer Sequences / Assay ID** | **Efficiency** |
| --- | --- | --- | --- | --- |
| Reference  Gene | *18S* | SYBR Green | f—CGG ACA GGA TTG ACA GAT TGA TAG  r—TGC CAG AGT CTC GTT CGT TA | 92% |
|  |  | TaqMan | Hs03003631_g1 | - |
| NP catabolic  genes | *COX2* | SYBR Green | f—GTC TGG TGC CTG GTC TGA  r—GTC TGG AAC AAC TGC TCA TCA C | 96% |
|  | *IL6* | TaqMan | Hs00174131_m1 |  |
| ECM modulators /  Mechanosensory | *MMP3* | SYBR Green | f—CAA GGC ATA GAG ACA ACA TAG A  r—GCA CAG CAA CAG TAG GAT | 78% |
|  |  | TaqMan | Hs00968305_m1 | - |
|  | *TRPV4* | SYBR Green | f— GAT TCC TGC TCG TCT ACT TG  r— GTT GGT CTG GTC CTC ATT G | 108.9% |
| NP anabolic  genes | *ACAN* | SYBR Green | f— CAT CAC TGC AGC TGT CAC  r— AGC AGC ACT ACC TCC TTC | 89% |
|  |  | TaqMan | [Hs00153936_m1](https://www.thermofisher.com/taqman-gene-expression/product/Hs00153936_m1?CID=&ICID=&subtype=) | - |
|  | *COL2* | SYBR Green | f—AGC AGC AAG AGC AAG GAG AA  r—GTA GGA AGG TCA TCT GGA | 99% |
|  | *TIMP1* | SYBR Green | f—TCA ACC AGA CCA CCT TAT ACC A  r—ATC CGC AGA CAC TCT CCA T | 93% |

*18S: 18S ribosomal RNA; COX2: Cyclooxygenase 2b; IL6: Interleukin 6; MMP3: Metallopeptidase 3; TRVP4: Transient receptor potential cation channel subfamily V member 4; ACAN: Aggrecan; COL2: Collagen type 2; TIMP1: Metallopeptidase inhibitor 1. *Efficiency test performed in human placenta or NP cells.*
