# Supplementary material for: Ex Vivo and In Vitro Proteomic Approach to Elucidate the Relevance of IL‐4 and IL‐10 in Intervertebral Disc Pathophysiology
Source: JOR Spine. 2025 Feb 10;8(1):e70048. doi: 10.1002/jsp2.70048 (PMC11808320; doi:10.1002/jsp2.70048)
Supplement: Supplementary file 2 — Table S2. [file JSP2-8-e70048-s001.docx]

**Table S2**: Soluble secretome protein panels used by luminex-based ELISA assays

|  | **Gene name** | **Cytokine** | | **Uniprot ID** | | **Protein Name** | | **LOD (pg/mL)** | | **LLOQ (pg/mL)** | | **ULOQ (pg/mL)** | |  |
| --- | --- | --- | --- | --- | --- | --- | --- | --- | --- | --- | --- | --- | --- | --- |
| **PANEL 1** | IL4 | IL4 | | P05112 | | Interleukin-4 | | 23 | | 23 | | 5500 | |  |
|  | IL1B | IL1b | | P01584 | | Interleukin-1 beta | | 1 | | 3 | | 1900 | |  |
|  | IL1A | IL1A | | P01583 | | Interleukin-1 alpha | | 2 | | 3 | | 1900 | |  |
|  | IFNG | IFNG | | P01579 | | Interferon gamma | | 10 | | 10 | | 5500 | |  |
|  | CSF3R | G-CSF | | P09919 | | Granulocyte colony-stimulating factor | | 32 | | 32 | | 5500 | |  |
|  | SCF | SCF | | P21583 | | Kit ligand | | 1 | | 1 | | 600 | |  |
|  | IL17F | IL17F | | Q96PD4 | | Interleukin-17F | | 21 | | 23 | | 16600 | |  |
|  | IL7 | IL7 | | P13232 | | Interleukin-7 | | 2 | | 3 | | 5500 | |  |
|  | IL16 | IL16 | | Q14005 | | Interleukin-16 | | 2 | | 3 | | 5500 | |  |
|  | IL18 | IL18 | | Q14116 | | Interleukin-18 | | 0 | | 1 | | 1900 | |  |
|  | IL6 | IL6 | | P05231 | | Interleukin-6 | | 0 | | 1 | | 1900 | |  |
|  | PROK1 | PROK1 | | Q9HC23 | | Prokineticin-1 | | 4 | | 8 | | 16600 | |  |
|  | TNFA | TNFA | | P01375 | | Tumor necrosis factor | | 1 | | 3 | | 5500 | |  |
|  | IL20 | IL20 | | Q9NYY1 | | Interleukin-20 | | 2 | | 8 | | 16600 | |  |
|  | FGF2 | FGF BASIC | | P09038 | | Fibroblast growth factor 1 | | 6 | | 8 | | 5500 | |  |
|  | IL10 | IL10 | | P22301 | | Interleukin-10 | | 0 | | 3 | | 5500 | |  |
|  | IL1RA | IL1RA | | P18510 | | Interleukin-1 receptor antagonist protein | | 23 | | 23 | | 5500 | |  |
| **PANEL 2** | CCL19 | CCL19 | | P78556 | | C-C motif chemokine 19 | | 40 | | 99 | | 8000 | |  |
|  | CXCL13 | CXCL13 | | O43927 | | C-X-C motif chemokine 13 | | 70 | | 70 | | 16600 | |  |
|  | CXCL12 | CXCL12 | | P48061 | | Stromal cell-derived factor 1 | | 193 | | 206 | | 50000 | |  |
|  | CSF2 | GM-CSF | | P04141 | | Granulocyte-macrophage colony-stimulating factor | | 0 | | 1 | | 3200 | |  |
|  | CSF1R | M-CSF | | P09603 | | Macrophage colony-stimulating factor 1 | | 1 | | 1 | | 3200 | |  |
|  | IL13 | IL13 | | P35225 | | Interleukin-13 | | 10 | | 11 | | 8000 | |  |
|  | IL2RA | IL2RA | | P01589 | | Interleukin-2 receptor subunit alpha | | 57 | | 57 | | 50000 | |  |
|  | IL15 | IL15 | | P40933 | | Interleukin-15 | | 0 | | 1 | | 1300 | |  |
|  | LIF | LIF | | P15018 | | Leukemia inhibitory factor | | 8 | | 8 | | 5500 | |  |
|  | IFNA2 | IFNA2 | | P01563 | | Interferon alpha-2 | | 1 | | 1 | | 3200 | |  |
|  | IL12 | IL12 | | P29459 | | Interleukin-12 subunit alpha | | 4 | | 4 | | 5500 | |  |
|  | IL11 | IL11 | | P20809 | | Interleukin-11 | | 122 | | 122 | | 20000 | |  |
|  | IL22 | IL22 | | Q9GZX6 | | Interleukin-22 | | 1 | | 1 | | 3200 | |  |
|  | CCL27 | CTACK | | Q9Y4X3 | | C-C motif chemokine 27 | | 13 | | 23 | | 5500 | |  |
|  | CXCL10 | IP10 | | P02778 | | C-X-C motif chemokine 10, 10 kDa interferon gamma-induced protein | | 0 | | 1 | | 3200 | |  |
|  | IL9 | IL9 | | P15248 | | Interleukin-9 | | 3 | | 3 | | 5500 | |  |
|  | IL17A | IL17A | | Q16552 | | Interleukin-17A | | 6 | | 6 | | 5500 | |  |
|  | CCL2 | CCL2 | | P13500 | | C-C motif chemokine 2, Monocyte chemotactic protein 1 | | 3 | | 3 | | 1900 | |  |
| **PANEL 3** | TNFRSF9 | TNFRSF9 | | P41273 | | Tumor necrosis factor receptor superfamily member 9 | | 68 | | 617 | | 16700 | |  |
|  | S100A8 | S100A8 | | P05109 | | Protein S100-A8 | | 17 | | 23 | | 50000 | |  |
|  | TNF10 | TNF10 | | P50591 | | Tumor necrosis factor ligand superfamily member 10 | | 0 | | 1 | | 1900 | |  |
|  | FST | FST | | P19883 | | Follistatin | | 37 | | 37 | | 5500 | |  |
|  | NGF | NGF | | P01138 | | Beta-nerve growth factor | | 6 | | 205 | | 5500 | |  |
|  | MMP13 | MMP13 | | P45452 | | Collagenase 3 | | 0 | | 1 | | 1900 | |  |
|  | ST2 | ST2 | | Q9UBE8 | | Interleukin-1 receptor-like 1 | | 65 | | 370 | | 10000 | |  |
|  | NRG1 | NRG1 | | Q02297 | | Pro-neuregulin-1, membrane-bound isoform | | 1 | | 2 | | 1900 | |  |
|  | RETN | RETN | | Q9HD89 | | Resistin | | 8 | | 205 | | 16600 | |  |
|  | CNTF | CNTF | | P26441 | | Ciliary neurotrophic factor | | 2 | | 3 | | 5500 | |  |
|  | MMP1 | MMP1 | | P03956 | | Interstitial collagenase | | 0 | | 0 | | 210 | |  |
| **PANEL 4** | CCL20 | CCL20 | | P78556 | | C-C motif chemokine 20 | | 2 | | 3 | | 5500 | |  |
|  | MIF | MIF | | P14174 | | Macrophage migration inhibitory factor | | 8 | | 23 | | 50000 | |  |
|  | CXCL1 | GROA | | P09341 | | Growth-regulated alpha protein | | 7 | | 8 | | 16600 | |  |
|  | MCP3 | CCL7 | | P80098 | | C-C motif chemokine 7 | | 17 | | 68 | | 16600 | |  |
|  | CXCL16 | CXCL16 | | O95799 | | C-X-C motif chemokine 16 | | 20 | | 20 | | 16600 | |  |
|  | CCL17 | CCL17 | | Q92583 | | C-C motif chemokine 17 | | 2 | | 3 | | 5500 | |  |
|  | CCL22 | CCL22 | | O00626 | | C-C motif chemokine 22 | | 7 | | 23 | | 5500 | |  |
|  | CCL3 | CCL3 | | P10147 | | C-C motif chemokine 3, Macrophage inflammatory protein 1-alpha | | 3 | | 23 | | 50000 | |  |
|  | CXCL11 | CXCL11 | | O14625 | | C-X-C motif chemokine 11, Interferon-inducible T-cell alpha chemoattractant | | 4 | | 8 | | 16600 | |  |
|  | TNF12 | TWEAK | | O43508 | | Tumor necrosis factor ligand superfamily member 12 | | 3 | | 8 | | 16600 | |  |
|  | CXCL9 | CXCL9 | | Q07325 | | C-X-C motif chemokine 9 | | 8 | | 8 | | 5500 | |  |
| **PANEL 5** | CCL4 | CCL4 | | P13236 | | C-C motif chemokine 4 | | 2 | | 2 | | 67 | |  |
|  | TNFSF11 | | sRANK-L | | O14788 | | Tumor necrosis factor ligand superfamily member 11 | | 3 | | 3 | | 5500 | |
|  | VEGF | | VEGF | | P15692 | | Vascular endothelial growth factor A | | 7 | | 23 | | 50000 | |
|  | IL8 | | IL8 | | P10145 | | Interleukin-8 | | 1 | | 1 | | 620 | |
|  | IL5 | | IL5 | | P05113 | | Interleukin-5 | | 1 | | 1 | | 1850 | |
|  | MMP7 | | MMP7 | | P09237 | | Matrilysin | | 4 | | 4 | | 5500 | |
|  | TGFB1 | | TGF-b1 | | P01137 | | Transforming growth factor beta-1 protein | | 2 | | 68 | | 16600 | |
|  | CCL11 | | Eotaxin | | P51671 | | Eotaxin | | 2 | | 3 | | 5500 | |
| **PANEL 6** | VCAM1 | | VCAM1 | | P19320 | | Vascular cell adhesion protein 1 | | 1 | | 1 | | 1900 | |
|  | TIMP1 | | TIMP1 | | P01033 | | Metalloproteinase inhibitor 1 | | 1 | | 1 | | 1900 | |
|  | MMP2 | | MMP2 | | P08253 | | 72 kDa type IV collagenase | | 8 | | 8 | | 16600 | |
|  | MMP9 | | MMP9 | | P14780 | | Matrix metalloproteinase-9 | | 3 | | 3 | | 5500 | |
|  | CCL5 | | RANTES | | P13501 | | C-C motif chemokine 5 | | 2 | | 3 | | 5500 | |
|  | ICAM1 | | ICAM1 | | P05362 | | Intercellular adhesion molecule 1 | | 0 | | 1 | | 1900 | |
|  | SERPINE1 | | PAI-1 | | P05121 | | Plasminogen activator inhibitor 1 | | 3 | | 3 | | 5500 | |
|  | DEFB1 | | DEFB1 | | P60022 | | Beta-defensin 1 | | 1 | | 3 | | 600 | |

Abbreviations: LOD, Limit of detection; LLOQ, Low limit of quantification; ULOQ, Upper limit of quantification
