# Supplementary material for: Ex Vivo and In Vitro Proteomic Approach to Elucidate the Relevance of IL‐4 and IL‐10 in Intervertebral Disc Pathophysiology
Source: JOR Spine. 2025 Feb 10;8(1):e70048. doi: 10.1002/jsp2.70048 (PMC11808320; doi:10.1002/jsp2.70048)
Supplement: Supplementary file 3 — Table S3. [file JSP2-8-e70048-s002.docx]

**Table S3**: Intracellular phophorylated protein panels used by luminex-based ELISA assays

|  | **Gene name** | **Protein** | **Uniprot ID** | **Phosphorylated residue** | **Sample/Noise** | **Pass/Fail** |
| --- | --- | --- | --- | --- | --- | --- |
| **PHOSPHO PANEL 1** | SMAD3 | SMAD3 | P84022 | S423/S425 | 2.83 | pass |
|  | TP53 | P53 | P04637 | S15 | 23.91 | pass |
|  | CREB1 | CREB1 | P16220 | S133 | 45.48 | pass |
|  | AKT1S1 | AKT1S1 | Q96B36 | T246 | 162.23 | pass |
|  | NFKBIA | IKBA | P25963 | S32/S36 | 5.08 | pass |
|  | PTK2 | FAK | Q05397 | Y397 | 3.92 | pass |
|  | GSK3A | GSK3 | P49840 | S21 | 16.65 | pass |
|  | AKT1 | AKT1 | P31749 | S473 | 18.68 | pass |
|  | HSPB1 | HSPB1 | P04792 | S78/S82 | 531.63 | pass |
|  | RPP38 | P38 | P78345 | T180/Y182 | 47.75 | pass |
|  | MTOR | mTOR | P42345 | S2448 | 3.36 | pass |
|  | MAP3K1 | MEK1 | Q13233 | S217/S221 | 79.79 | pass |
|  | RPS6KA1 | RSK1 | Q15418 | S380 | 36.02 | pass |
| **PHOSPHO PANEL 2** | JUN | JUN | P05412 | S63 | 83.12 | pass |
|  | P00533 | EGFR | P00533 | Y1068 | 109.78 | pass |
|  | MAPK3 | ERK1 | P27361 | T202 | 128.29 | pass |
|  | MARCKSL1 | MARCKS | P49006 | S170 | 74.88 | pass |
|  | PTPN11 | PTN11 | Q06124 | Y542 | 85.95 | pass |
|  | CHEK2 | CHK2 | O96017 | T68 | 1.79 | pass |
|  | STAT3 | STAT3 | P40763 | Y705 | 4.59 | pass |
|  | NFKB1 | NFKB | P19838 | S536 | 114.61 | pass |
