# Supplementary material for: Ex Vivo and In Vitro Proteomic Approach to Elucidate the Relevance of IL‐4 and IL‐10 in Intervertebral Disc Pathophysiology
Source: JOR Spine. 2025 Feb 10;8(1):e70048. doi: 10.1002/jsp2.70048 (PMC11808320; doi:10.1002/jsp2.70048)
Supplement: Supplementary file 4 — Table S4. [file JSP2-8-e70048-s005.docx]

**Table S4**: Signal and noise controls for phosphoproteomic measurements.

| **Stimulated controls and noise controls** | **SMAD3** | **P53** | **CREB1** | **AKT1S1** | **IKBA** | **FAK** | **GSK3** | | **AKT1** | **HSPB1** | **P38** | **MTOR** | **MEK1** | | | **RSK1** | **JUN** | **EGFR** | | **ERK1** | **MARCKS** | **PTN11** | | **CHK2** | | **STAT3** | | **NFKB** | |
| --- | --- | --- | --- | --- | --- | --- | --- | --- | --- | --- | --- | --- | --- | --- | --- | --- | --- | --- | --- | --- | --- | --- | --- | --- | --- | --- | --- | --- | --- |
| **231_pervanadate** | 114.50 | 1330.50 | 8245.50 | 5571.00 | 88.50 | 159.00 | 444.50 | 2431.00 | | 142.00 | 2763.00 | 122.50 | | 5691.0 | 453.00 | | 371.50 | 308.00 | 6949.50 | | 2471.00 | 3610.0 | 50.00 | | 247.00 | | 852.00 | |  |
| **231_phosphatase** | 69.00 | 136.00 | 339.00 | 39.00 | 58.50 | 40.50 | 105.00 | 131.00 | | 64.00 | 514.50 | 42.00 | | 314.50 | 97.00 | | 42.00 | 51.00 | 2800.00 | | 33.00 | 42.00 | 46.00 | | 87.00 | | 87.00 | |  |
| **S/N** | 1.66 | 9.78 | 24.32 | 142.85 | 1.51 | 3.93 | 4.23 | 18.56 | | 2.22 | 5.37 | 2.92 | | 18.10 | 4.67 | | 8.85 | 6.04 | 2.48 | | 74.88 | 85.95 | 1.09 | | 2.84 | | 9.79 | |  |
| **HEK293_TNFA-Cal** | 102.00 | 229.00 | 12190.50 | 4218.00 | 41.00 | 49.00 | 699.50 | 1703.00 | | 357.00 | 2178.00 | 50.00 | | 936.00 | 419.00 | | 2327.00 | 24.00 | 480.50 | | 1556.50 | 85.00 | 42.00 | | 118.50 | | 5616.0 | |  |
| **HEK293_Phosphatase** | 56.50 | 35.50 | 268.00 | 26.00 | 27.00 | 31.00 | 42.00 | 196.50 | | 23.00 | 319.50 | 30.00 | | 41.00 | 31.00 | | 28.00 | 15.00 | 38.50 | | 38.00 | 40.00 | 34.00 | | 32.00 | | 49.00 | |  |
| **S/N** | 1.81 | 6.45 | 45.49 | 162.23 | 1.52 | 1.58 | 16.65 | 8.67 | | 15.52 | 6.82 | 1.67 | | 22.83 | 13.52 | | 83.11 | 1.60 | 12.48 | | 40.96 | 2.13 | 1.24 | | 3.70 | | 114.61 | |  |
| **HEK293_UV** | 73.00 | 179.00 | 3318.00 | 4147.00 | 40.00 | 28.00 | 249.00 | 1542.00 | | 27.50 | 350.00 | 45.00 | | 174.50 | 44.00 | | 640.50 | 14.50 | 146.00 | | 1026.00 | 41.00 | 23.00 | | 80.50 | | 164.50 | |  |
| **HEK293_Phosphatase** | 56.50 | 35.50 | 268.00 | 26.00 | 27.00 | 31.00 | 42.00 | 196.50 | | 23.00 | 319.50 | 30.00 | | 41.00 | 31.00 | | 28.00 | 15.00 | 38.50 | | 38.00 | 40.00 | 34.00 | | 32.00 | | 49.00 | |  |
| **S/N** | 1.29 | 5.04 | 12.38 | 159.50 | 1.48 | 0.90 | 5.93 | 7.85 | | 1.20 | 1.10 | 1.50 | | 4.26 | 1.42 | | 22.88 | 0.97 | 3.79 | | 27.00 | 1.03 | 0.68 | | 2.52 | | 3.36 | |  |
| **HELA_TNFA-Cal** | 62.00 | 31.50 | 3124.00 | 2915.00 | 39.00 | 38.50 | 178.00 | 563.50 | | 2519.00 | 1046.00 | 31.00 | | 3431.0 | 304.50 | | 69.50 | 28.00 | 354.50 | | 759.00 | 33.00 | 34.00 | | 59.00 | | 1493.0 | |  |
| **HELA_Phosphatase** | 50.00 | 29.50 | 281.00 | 28.00 | 25.00 | 25.00 | 40.00 | 34.00 | | 9.50 | 112.00 | 40.00 | | 43.00 | 30.00 | | 41.00 | 12.00 | 32.50 | | 29.00 | 34.50 | 22.50 | | 34.00 | | 50.00 | |  |
| **S/N** | 1.24 | 1.07 | 11.12 | 104.11 | 1.56 | 1.54 | 4.45 | 16.57 | | 265.16 | 9.34 | 0.78 | | 79.79 | 10.15 | | 1.70 | 2.33 | 10.91 | | 26.17 | 0.96 | 1.51 | | 1.74 | | 29.86 | |  |
| **HEK293_H2O2** | 86.00 | 155.50 | 3712.00 | 1156.00 | 43.00 | 36.00 | 182.00 | 1657.00 | | 35.00 | 1964.00 | 44.00 | | 141.00 | 103.00 | | 247.00 | 22.00 | 125.00 | | 46.00 | 41.50 | 61.00 | | 82.50 | | 228.00 | |  |
| **HEK293_Phosphatase** | 56.50 | 35.50 | 268.00 | 26.00 | 27.00 | 31.00 | 42.00 | 196.50 | | 23.00 | 319.50 | 30.00 | | 41.00 | 31.00 | | 28.00 | 15.00 | 38.50 | | 38.00 | 40.00 | 34.00 | | 32.00 | | 49.00 | |  |
| **S/N** | 1.52 | 4.38 | 13.85 | 44.46 | 1.59 | 1.16 | 4.33 | 8.43 | | 1.52 | 6.15 | 1.47 | | 3.44 | 3.32 | | 8.82 | 1.47 | 3.25 | | 1.21 | 1.04 | 1.79 | | 2.58 | | 4.65 | |  |
| **Hela _cell lysate_TNFa_Calyculin A**  **(Millipore)_ 200ug/mL** | 107.00 | 46.00 | 481.00 | 826.00 | 127.0 | 70.00 | 286.50 | 635.00 | | 5050.50 | 5348.50 | 134.50 | | 2245.0 | 1080.5 | | 86.00 | 25.00 | 4169.50 | | 717.00 | 51.00 | 36.00 | | 156.00 | | 3225.0 | |  |
| **HELA_Phosphatase** | 50.00 | 29.50 | 281.00 | 28.00 | 25.00 | 25.00 | 40.00 | 34.00 | | 9.50 | 112.00 | 40.00 | | 43.00 | 30.00 | | 41.00 | 12.00 | 32.50 | | 29.00 | 34.50 | 22.50 | | 34.00 | | 50.00 | |  |
| **S/N** | 2.14 | 1.56 | 1.71 | 29.50 | 5.08 | 2.80 | 7.16 | 18.68 | | 531.63 | 47.75 | 3.36 | | 52.21 | 36.02 | | 2.10 | 2.08 | 128.29 | | 24.72 | 1.48 | 1.60 | | 4.59 | | 64.50 | |  |
| **EGF-Treated_HELA _(Biorad)_ 200ug/mL** | 141.50 | 39.00 | 1355.50 | 110.00 | 54.00 | 86.00 | 239.00 | 266.50 | | 47.50 | 772.50 | 49.00 | | 2113.0 | 239.00 | | 49.00 | 34.50 | 2085.50 | | 166.00 | 54.00 | 38.00 | | 99.50 | | 506.50 | |  |
| **HELA_Phosphatase** | 50.00 | 29.50 | 281.00 | 28.00 | 25.00 | 25.00 | 40.00 | 34.00 | | 9.50 | 112.00 | 40.00 | | 43.00 | 30.00 | | 41.00 | 12.00 | 32.50 | | 29.00 | 34.50 | 22.50 | | 34.00 | | 50.00 | |  |
| **S/N** | 2.83 | 1.32 | 4.82 | 3.93 | 2.16 | 3.44 | 5.98 | 7.84 | | 5.00 | 6.90 | 1.23 | | 49.14 | 7.97 | | 1.20 | 2.88 | 64.17 | | 5.72 | 1.57 | 1.69 | | 2.93 | | 10.13 | |  |
| **A431 cell lysate_ EGF (Millipore)_ 200ug/mL** | 116.00 | 1124.00 | 1209.00 | 471.00 | 105.50 | 127.00 | 269.00 | 543.00 | | 78.00 | 524.00 | 67.00 | | 2558.5 | 964.00 | | 104.0 | 2964.0 | 6354.00 | | 85.00 | 195.00 | 45.00 | | 248.50 | | 786.50 | |  |
| **Non-Stimulated_A431_Cell_**  **Lysate_(Millipore)_200ug_mL** | 73.50 | 47.00 | 817.00 | 36.00 | 124.00 | 38.00 | 45.50 | 46.00 | | 32.00 | 729.00 | 58.00 | | 222.00 | 785.00 | | 195.0 | 27.00 | 2937.50 | | 48.00 | 28.50 | 34.00 | | 67.50 | | 115.00 | |  |
| **S/N** | 1.58 | 23.91 | 1.48 | 13.08 | 0.85 | 3.34 | 5.91 | 11.80 | | 2.44 | 0.72 | 1.16 | | 11.52 | 1.23 | | 0.53 | 109.78 | 2.16 | | 1.77 | 6.84 | 1.32 | | 3.68 | | 6.84 | |  |
| **Average S/N** | 2.83 | 23.91 | 45.49 | 162.23 | 5.08 | 3.93 | 16.65 | 18.68 | | 531.63 | 47.75 | 3.36 | | 79.79 | 36.02 | | 83.11 | 109.78 | 128.29 | | 74.88 | 85.95 | 1.79 | | 4.59 | | 114.61 | |  |

Signal and noise values are reported as Mean Fluorescence Intesities (MFI).Signal/noise ratios relevant for each phosphorylated protein is highlighted in green
